# Novel use of a servosphere to study apodous insects: Investigation of blow fly post‐feeding larval dispersal

**DOI:** 10.1111/mve.12745

**Published:** 2024-07-23

**Authors:** Molly Mactaggart, Amoret P. Whitaker, Keith N. Wilkinson, Martin J. R. Hall

**Affiliations:** ^1^ Faculty of Law, Crime and Justice University of Winchester Winchester UK; ^2^ Natural History Museum London UK

**Keywords:** Calliphoridae, forensic entomology, negative phototaxis, post‐feeding dispersal, servosphere

## Abstract

Blow flies (Diptera: Calliphoridae) are arguably the most important providers of an estimate of minimum post‐mortem interval in forensic investigations. They usually undergo a post‐feeding dispersal from the body. While previous studies have looked at dispersal of groups of larvae, recording the dispersal activity of individual larvae has not previously been demonstrated. A servosphere was used here to record the speed, directionality and phototaxis of individual post‐feeding larvae of two species of blow fly on a smooth plastic surface over time. The servosphere rotates to compensate for the movement of an insect placed at its apex, thereby enabling its unimpeded locomotion in any direction to be studied and behavioural changes to external stimuli recorded. To our knowledge, the servosphere has not previously been used to study apodous insects. The objective of our study was to compare dispersal behaviour of *Calliphora vicina* Robineau‐Desvoidy and *Protophormia terraenovae* (Robineau‐Desvoidy), both common primary colonisers of human and animal cadavers, but showing different post‐feeding dispersal strategies. Larvae of *C. vicina* generally disperse from the body while those of *P. terraenovae* remain on or close to the body. Our aims were to study (1) changes in dispersal speed over a 1‐h period; (2) changes in dispersal speed once a day for 4 days, between the end of feeding and onset of pupariation; and (3) response of dispersing larvae to light. We demonstrated that (1) the movement of three *C. vicina* larvae tracked for 1 continuous hour on 1 day slowed from an average of 3 to <1.7 mms^−1^; (2) the average speed of 20 larvae of *C. vicina* (4.08 mms^−1^) recorded for 5 min once per day over a 4‐day period between onset of dispersal and pupariation was significantly greater than that of *P. terraenovae* (2.36 mms^−1^; *p* < 0.0001), but that speed of both species increased slightly over the 4 days; (3) the responses of larvae of *C. vicina* to changes in light direction from the four cardinal directions of the compass, showed that they exhibited a strong negative phototactic response within 5 s, turning to move at approximately 180° away from the new light position. While conducted to observe larval calliphorid post‐feeding behaviour, the results of this proof of concept study show that apodous insects can be studied on a servosphere to produce both qualitative and quantitative data.

## Graphical abstract

- A servosphere was used to record the speed, directionality and phototaxis of individual post‐feeding larvae of two species of blow fly (Diptera: Calliphoridae) on a smooth plastic surface over time.
- The servosphere rotates to compensate for the movement of an insect placed at its apex, thereby facilitating its unimpeded locomotion in any direction and enabling behavioural changes to external stimuli to be recorded.
- The results of this proof‐of‐concept study show that apodous insects can be studied on a servosphere to produce both qualitative and quantitative data.

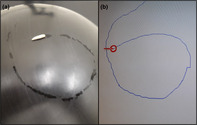

## INTRODUCTION

The servosphere, originally designed by Ernst Kramer (Kramer, 1976), is an apparatus that enables observation of the walking behaviour of podous insects. The servosphere rotates to compensate for the movement of an insect placed at its apex, and displacements of the sphere generated by the insect's movement can be measured at an interval of 0.1 s with an accuracy of 0.1 mm. Through this mechanism, the unimpeded walking behaviour of an insect can be studied and any behavioural changes to external stimuli recorded (Arnold et al., 2016). Insects that have been studied using this apparatus include Coleoptera (McMahon & Guerin, 2000; Otálora‐Luna & Dickens, 2011; von Hoermann et al., 2013), Hemiptera (Otalora‐Luna et al., 2004; Taneja & Guerin, 1995), Blattodea (Bell & Kramer, 1980) and Lepidoptera (Sakuma, 2002). Other arthropod groups that are not insects have also been studied, such as Acariformes (Rickli et al., 1992) and Isopoda (Nagaya et al., 2017). Moreover, new servosphere tracking techniques are possible for some insects, especially long‐bodied species, with new software developed (Yasushi et al., 2019).

The servosphere experiments conducted here were carried out to examine aspects of the post‐feeding larval dispersal of blow flies (Diptera: Calliphoridae) under controlled environmental conditions. Studies of larval dispersal are important in forensic entomology in order to determine where dispersed larvae, and subsequent puparia, are located—if these types of forensic evidence are overlooked then an underestimation of minimum post‐mortem interval is likely (Gomes et al., 2006; Lewis & Benbow, 2011; Turpin et al., 2014). Dispersing larvae are readily able to burrow into a friable substrate, such as soil, prior to pupariation (Gomes et al., 2005), but on a solid substrate, such as indoor floors, they cannot burrow and so continue to move for longer (Robinson et al., 2018). *Calliphora vicina* Robineau‐Desvoidy was the primary species studied here because it is known to be one of the most common calliphorid species in the UK and Europe to colonise human remains (Arnott & Turner, 2008; Baqué et al., 2015; Hodecek et al., 2024). *Protophormia terraenovae* (Robineau‐Desvoidy) is also a common UK species and is known to generally remain close to, or indeed on, the body after feeding (Arnott & Turner, 2008; Byrd & Castner, 2009; Erzinçlioğlu, 1996; Pohjoismäki et al., 2010) and was, therefore, identified as an appropriate species to provide data for comparison with *C. vicina*, which does undergo post‐feeding larval dispersal (Arnott & Turner, 2008), lasting from around 2–7 days depending on temperature (Anderson, 2000).

The main factors of post‐feeding larval dispersal that were studied were speed and total distance dispersed. It has been demonstrated that dispersal speed increases as a function of larval length for *P. terraenovae* (Charabidze et al., 2008). In laboratory colonies, fully developed larvae of *C. vicina* (16–18 mm; Bugelli et al., 2017; Salanitro et al., 2022) and *P. terraenovae* (17–18 mm, Grassberger & Reiter, 2002) are of similar size. Therefore, their dispersal speeds might also be expected to be similar (Charabidze et al., 2008). It might also be expected that there would be an overall decreasing speed over time from the onset of dispersal, due to a reduction in the available energy. Pupariation is initiated by the production of moulting hormones during the post‐feeding larval stage. One hormone known to be involved in this process is ecdysone, the concentrations of which are suppressed during the larval stages, but increase prior to pupariation (Beckstead et al., 2005; Thummel, 1996; Ward et al., 2003). There should be a decrease in the speed of post‐feeding larvae as the concentration of moulting hormones increases near pupariation, indeed hormonal factors that cause larval immobilisation were detected in the 1980s (Zdarek et al., 1981).

Calliphorid larvae possess light receptors (ocelli), which enable them to detect light (Byrd & Castner, 2009; Hinnemann et al., 2010). The post‐feeding larvae of at least some blow fly species, such as *Calliphora vomitoria* (L.) and *Lucilia caesar* (L.), tend to disperse at night (Kočárek, 2001). It is well known that post‐feeding larvae react negatively to the stimulus of light, as they are in search of a suitable pupariation site and, in an outdoor setting, this is often within the dispersal substrate itself (i.e., somewhere dark) (Benecke, 2005; Hinnemann et al., 2010). Negative phototaxis, however, has not been studied under the controlled conditions that are possible with the servosphere. Therefore, we also undertook a preliminary assessment of the responses of dispersing larvae to light stimuli.

## MATERIALS AND METHODS

Larvae were taken from established colonies of *C. vicina* and *P. terraenovae* that had been maintained at the Natural History Museum, London, for 24 months (*C. vicina*) and 3 months (*P. terraenovae*) before the study commenced. The colonies were originally established with *C. vicina* adults caught and refreshed at 3–6 month intervals using Redtop® fly traps (Miller Methods, Pretoria, South Africa) baited with pork liver, in the wildlife garden of the Natural History Museum (Ware et al., 2016) and wild *P. terraenovae* collected in Pontllanfraith village, Caerphilly County Borough, South Wales. The colonies were maintained at approximately 21°C and 60% relative humidity (RH) under a lighting cycle of 18:6 h (light:dark). Adults were fed sugar and water ad libitum and, when egg‐laying was required, also provided with milk powder and 2–4 mL of blood each day for a week, before being given pork liver as an oviposition substrate. Third instar larvae used for the study were collected within 12 h of when they first left the food source and were, therefore, identified as post‐feeding. Prior to collection, the larvae were provided with plentiful pork liver in order to mitigate starvation and early wandering behaviour.

**FIGURE 1:**
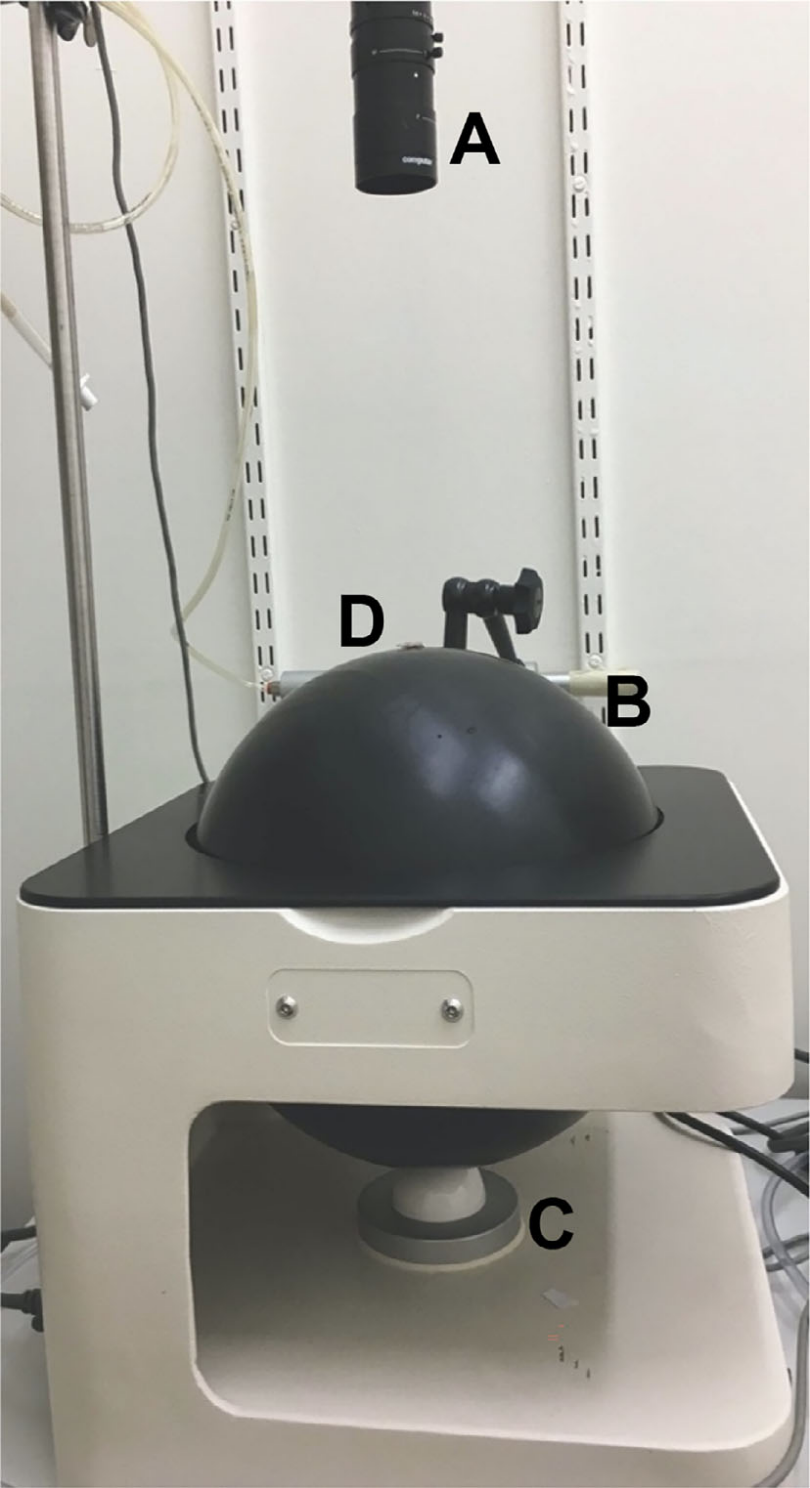
A photograph of the servosphere apparatus consisting of a CMOS camera (a), the servosphere sphere (b) the servomotor (c) and larval placement is shown at position (d). The lux metre was placed between (a) and (d). CMOS, complementary metal‐oxide semiconductor.

All experiments used a Syntech TrackSphere LC‐300 servosphere, consisting of the servosphere, a complementary metal‐oxide semiconductor camera and a control unit (Figure 1). Laboratory temperature and humidity were measured daily, at the start of the experiments, using a factory‐calibrated Tinytag Plus 2® datalogger: means (and ranges) were 21.5°C (21.4–22.2°C) and 38.9% RH (25.0%–45.0% RH). Lux light levels were measured daily using a data logging Heavy Duty Lightmeter (HD450) with the sensor aimed vertically upwards (Figure 1): mean (and range) was 412 lux (356–461 lux).

**FIGURE 2:**
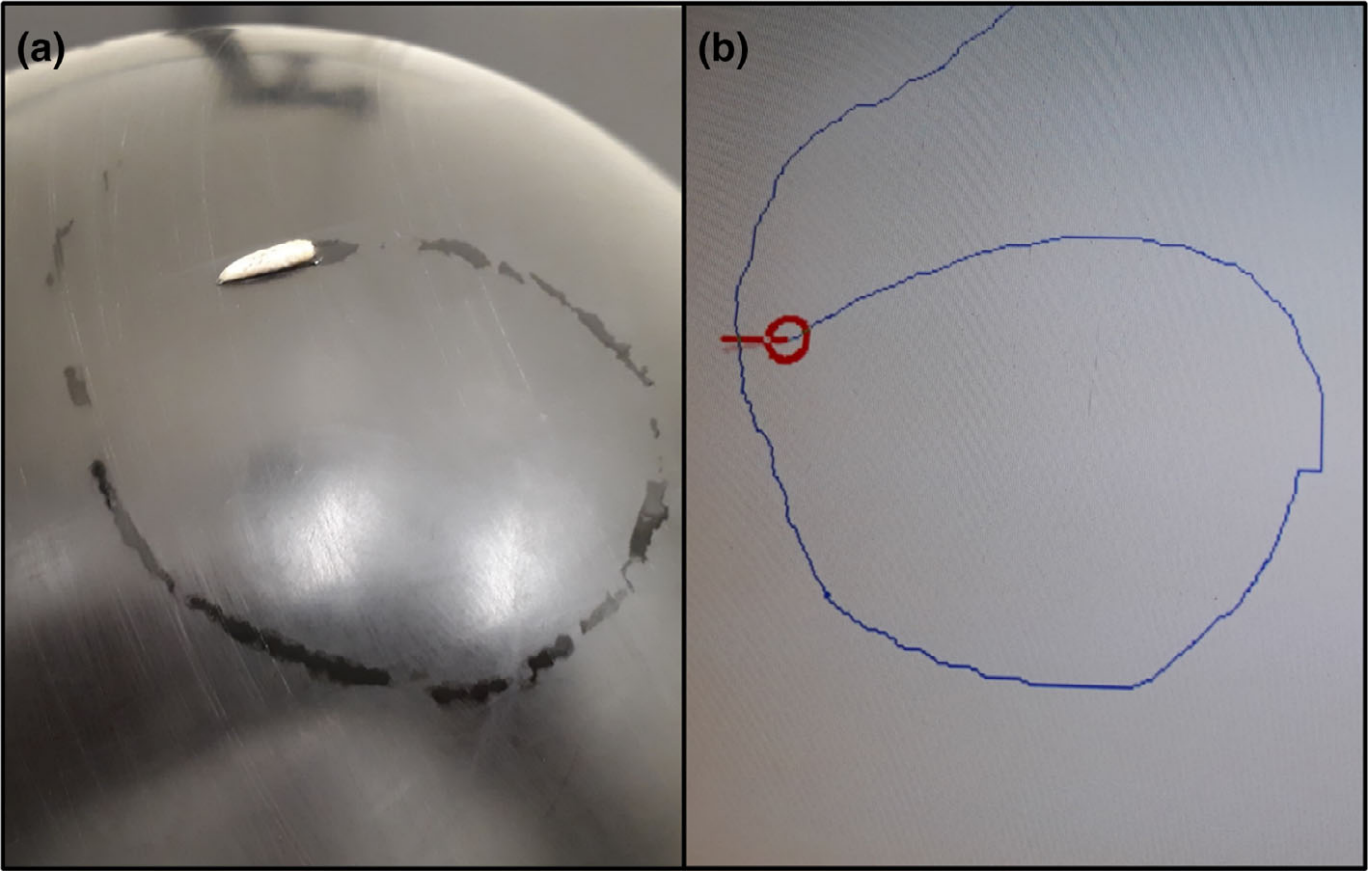
Photograph of a post‐feeding larva of *Calliphora vicina* (a) at the apex of the servosphere and a computer representation of the track of the same larva (b), captured just a few seconds after the photograph was taken.

For each recording session, a single post‐feeding larva was placed at the apex of the sphere and the camera tracked it, moving the servosphere through the control unit such that the larva's position was maintained at the apex. Larval tracks were recorded using Syntech TrackSphere 3.1 software, which provided raw and partially processed data, including coordinates of larval movements, track diagrams, average speeds and track lengths. After 1 min to acclimatise to moving on the sphere, the larva was gently moved with forceps so that it began the experiment facing east. Each larva was moistened with a drop of deionised water to ensure it retained traction on the sphere (see Videos S1 and S2 and Figure 2). The servosphere was cleaned between each larval run by wiping with 70% ethanol, to minimise the known potential for larvae to follow the trails of other larvae (Arnott & Turner, 2008). During the phototaxis experiments, the direction of the single light source was changed manually.

### Changes in dispersal speed over a 1‐h period

One post‐feeding *C. vicina* larva was used in each experiment and the experiment was repeated three times, each time with a different larva (*n* = 3). The average speed (mms^−1^) of each larva was recorded per second for 1 h. *Calliphora vicina* was the only species studied at this time due to the known dispersal behaviour of the species.

### Changes in dispersal speed over a 4‐day period between the end of feeding and start of pupariation

Ten post‐feeding larvae of *C. vicina* and 10 of *P. terraenovae* were studied for 5 min once a day, for 4 days. The same 20 specimens were used each day, unless they had pupariated (half of the *C. vicina*, *n* = 5, pupariated on the fourth day and half of the *P. terraenovae*, *n* = 5, pupariated on the second day). All larvae were kept at laboratory temperature for a 7‐h period each day, approximately 10:00–17:00 h. In order to slow development and delay pupariation, between daily runs the larvae were returned to a 1.3 L Drennan Maggibox with the other larvae of the same species, and stored at 3°C, a temperature that is above the lower development threshold for *C. vicina* (1°C; Donovan et al., 2006), but below that of *P. terraenovae* (9°C; Grassberger & Reiter, 2002).

### Responses of dispersing larvae to light

The responses of 12 post‐feeding *C. vicina* larvae to light were assessed, with the larvae facing east at the start of each run (Figure 1a). At the start of each experiment, all of the ceiling lights and other major light sources in the experimental room were turned off (a low light was still emitting from the computer screen, but as the lux readings recorded at the apex of the sphere were 0, it was decided that this would not interfere with the experiment). A larva was placed at the apex of the sphere and an external, hand‐held light source (iPhone torch) was then shone at the larva from approximately 30 cm, directly above. Once the larval movement was recognised by the camera (within 1–2 s), the position of the light was moved, manually, so that it shone at approximately 45° to the line of sight of the camera and the larva. The light source was moved for each of the 12 runs in one of four cardinal directions, north, east, south or west. Lux light levels of the light source were measured at the start of each experimental run using a data logging Heavy Duty Lightmeter (HD450); with the sensor aimed vertically upwards at the top of the sphere, the mean light level measured was 3000 lux (range 2250–3460 lux).

## RESULTS

### Changes in dispersal speed over a 1‐h period

**FIGURE 3:**
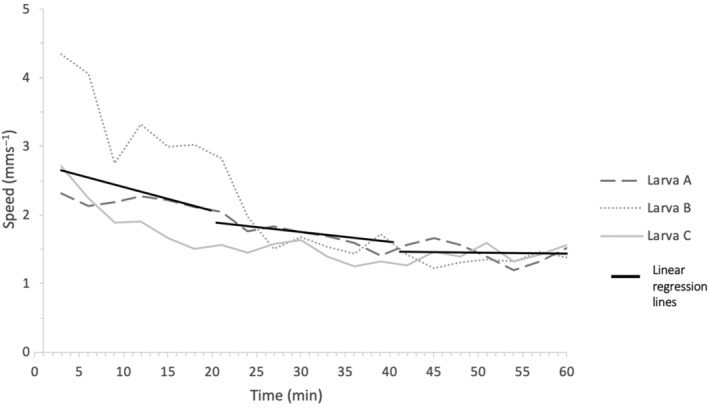
The change in the larval speed (mms^−1^) of each of three *Calliphora vicina* larva (a–c) over 1 h. The average speed (measured every second) during successive 3‐min periods was recorded at the end of each period. Three linear regression lines of the means over 20 min are fitted onto the graph (dashed lines), for 0–20, 20–40 and 40–60 min.

The movement of three *C. vicina* larvae tracked for 1 continuous hour on 1 day slowed from an average of 3 to <1.7 mms^−1^ (Figure 3). The gradients of the trendlines for the first, second and third 20‐min periods were −0.056, −0.018 and 0.0013, respectively (Figure 2), that is, larval speed decreased over time, until it was almost constant during the final 20 min. The mean distance dispersed by each larva over 1 h was 7.1 m (range, 5.8–7.7 m), equivalent to an average speed of 1.97 mms^−1^.

### Changes in dispersal speed over a 4‐day period between the end of feeding and pupariation

**FIGURE 4:**
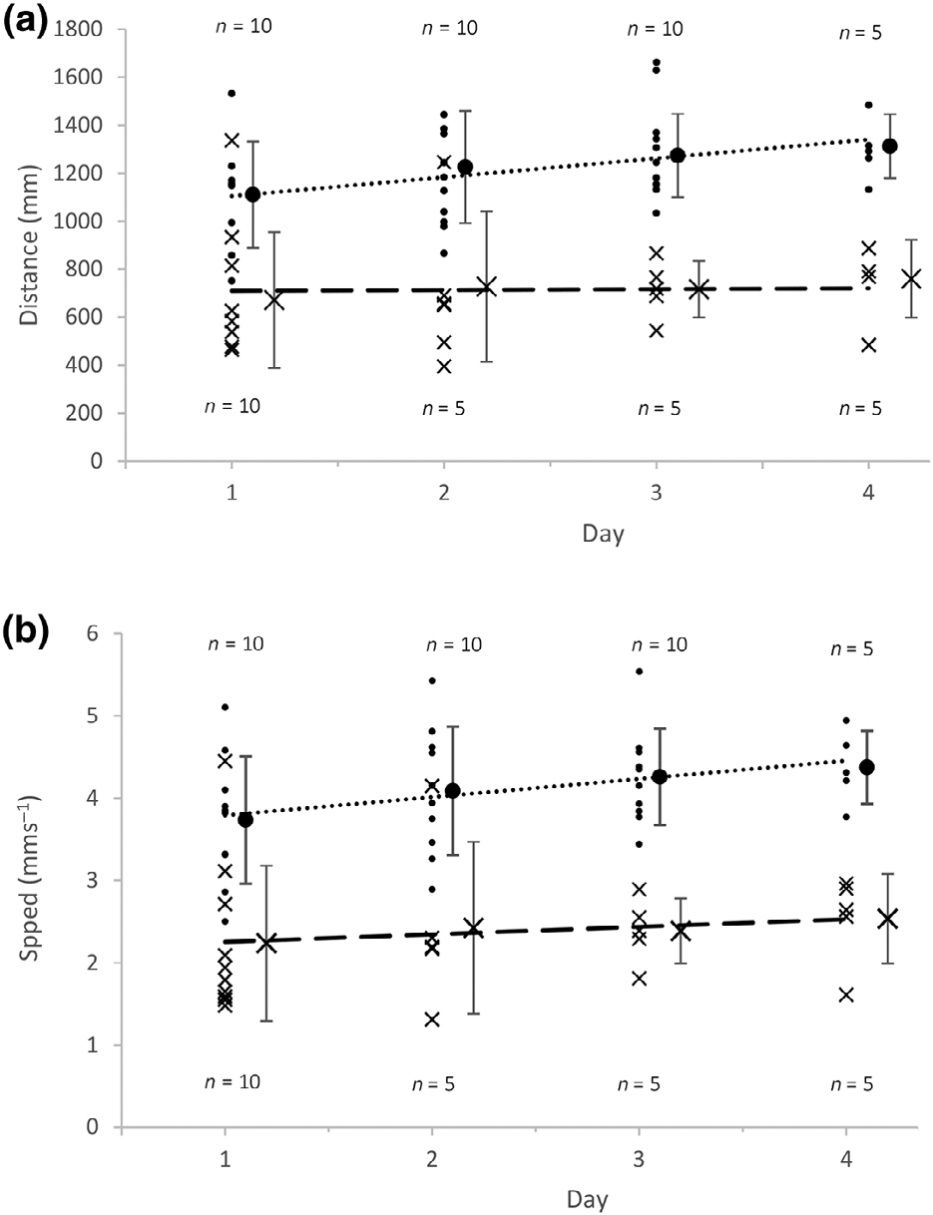
The overall track length (a) and average speed (b) of *C. vicina* (dots) and *P. terraenovae* (crosses) over 4 days. A linear regression line, based on individual larva data points, was fitted onto each graph for *C. vicina* (short dashes) and *P. terraenovae* (long dashes) (see Table 1). The length of each run each day was 5 min per larva. As some of the larvae pupariated, the values of n were 10 for *C. vicina* on days 1–3 and for *P. terraenovae* on day 1 and were 5 for *C. vicina* on day 4 and for *P. terraenovae* on days 2–4. The mean for each day for each species is shown with +/− standard deviation error bars, offset by +0.1 (*C. vicina*) or +0.2 (*P. terraenovae*) days for clarity.

The activity (speed and dispersal distance) of 20 larvae of *C. vicina* and *P. terraenovae* was recorded for 5 min once per day (approximately 10:00–17:00 h) over a 4‐day period after the onset of dispersal. Overall trend, for both species, showed a very slight increase in speed and overall track length over the 4‐day period (Figure 4). A *t*‐test was carried out in RStudio® to determine whether the slopes of the linear regression lines were significantly different to a gradient of 0 (Table 1). The slopes of the linear regression lines for both *C. vicina* and *P. terraenovae* were all significantly positive (*p* < 0.05), showing that the speed and distance dispersed of each species slightly, but statistically significantly, increased over the 4‐day period (Table 1). Over the 4 days, the average speed of *C. vicina* (4.08 mms^−1^) was significantly greater than that of *P. terraenovae* (2.36 mms^−1^; *p* < 0.0001). These average speeds equate to 14.7 mh^−1^ for *C. vicina* and 8.5 mh^−1^ for *P. terraenovae*. The average speed of *C. vicina* was higher here than in the 1‐h experiment, but it declined over the course of the 1‐h experiment from an initial average speed in the first 5 min of 2.97 mms^−1^.

**TABLE 1:** Statistical analysis (*t*‐tests) of the data presented in Figure 4, to determine whether the slopes of the linear regression lines were statistically significantly different to a gradient of 0.

Species	Data	Slope	*t* value	*p* value
*C. vicina*	Average speed	Positive	2.443	0.0201
*P. terraenovae*	Average speed	Positive	6.203	<0.0001
*C. vicina*	Track length	Positive	2.456	0.0195
*P. terraenovae*	Track length	Positive	6.143	<0.0001

### Responses of dispersing larvae to light

**FIGURES 5:**
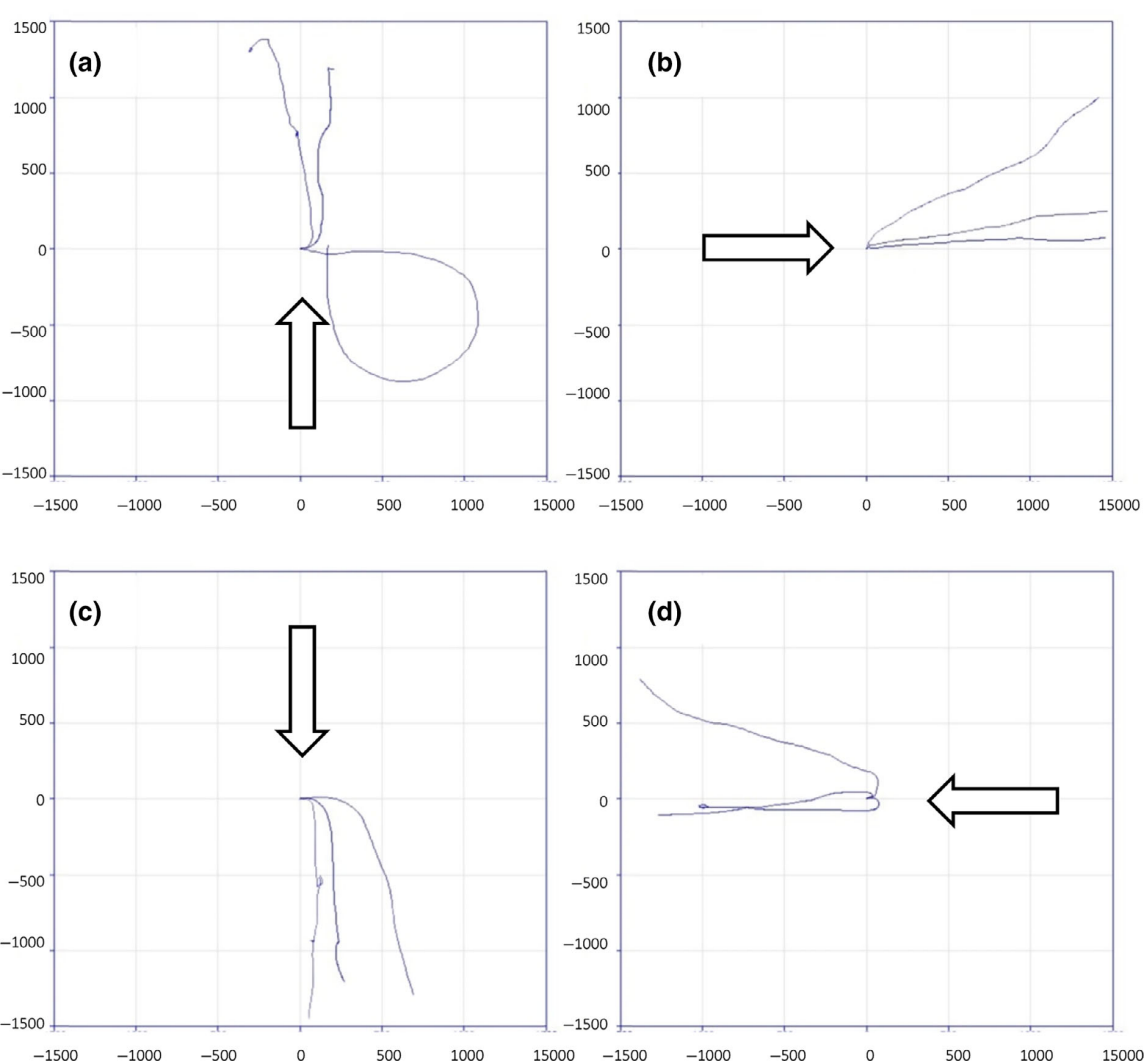
(a)–(d) Larval tracks of 12 *C. vicina* larvae. The tracks are shown on a 3000 × 3000 mm grid, where 0,0 was the origin of each larvae. Each larval run began with the larva facing east and light from overhead. A light source was then shone at 45° towards the larva from four different directions: north (a), east (b), south (c) and west (d), as indicated by the arrows. The length of each run was 5 min following change in light direction.

The responses of 12 larvae of *C. vicina* to changes in light direction from each of the four cardinal directions showed that they exhibited a strong negative phototactic response (Figure 5). Each larva was run for 5 min after the light change and, for 11 larvae, the initial negative phototaxis occurred within 5 s and was followed by continued larval movement directly away from the light source. Only one larva did not immediately move directly away from the light source, but its final direction of movement was approximately 180° away from the light, after approximately 2 min (Figure 5).

## DISCUSSION

The main limitation of this proof‐of‐concept study was clearly the low numbers of replicates, which allowed for only limited statistical analysis. Nevertheless, we demonstrated that the servosphere apparatus and recording system allowed for analysis of the movement of dispersing, third instar larvae of two calliphorid species in a controlled environment of temperature and humidity, without the spatial boundaries found in most oblong or circular arenas used for dispersal studies (Arnott & Turner, 2008; Gomes et al., 2007; Robinson et al., 2018). Temperature was the most important environmental variable to control as the speed of larval dispersal increases with temperature (Charabidze et al., 2008).

When post‐feeding calliphorid larvae disperse into a friable substrate, they rapidly burrow into the substrate prior to pupariation (Gomes et al., 2005). However, when dispersing on solid substrates, larvae are unable to burrow (Robinson et al., 2018) and continue to move until they pupariate under hormonal control (Zdarek et al., 1981). Our studies over 1‐h period show that larvae dispersing on solid surfaces decreased their speed gradually over the first 20 min to a plateau level, about 60% of the initial speed, but this is then maintained. Clearly, there is potential to extend these studies for longer time periods, but this was beyond the objectives and time limitations of this study.

It was hypothesised that as a larva nears pupariation the larva's speed would decrease as moulting hormones are produced initiating pupariation behaviour (Beckstead et al., 2005; Thummel, 1996). While the hormone ecdysone is suppressed during the larval stage, there is a spike in its production prior to pupariation as this hormone is known to be involved in the initiation of the pupariation stage of metamorphosis (Beckstead et al., 2005; Thummel, 1996; Ward et al., 2003). This study over a 4‐day period did not accurately reflect natural conditions, since for 17 h each day the larvae were stored at 3°C, between daily experiments at laboratory temperatures. This would slow larval development compared to a situation where the larvae were retained at ambient temperatures all day, even those with a natural fluctuation around the laboratory mean. Nevertheless, half of the original population, both of *C. vicina* and *P. terraenovae*, had pupariated by the end of the 4 days, showing that their development was progressing. Moreover, larvae of both of these species kept moving at a slightly increasing rate on the servosphere and continued to move inside their plastic containers between experimental runs, almost until pupariation. Of course, the larvae were only run once a day for 5 min and, therefore, between each larva's final run and their pupariation, there could have been up to 24 h during which time perhaps larval speed would have shown a decrease if tested. Future studies should examine change in larval speed at a finer time scale (e.g., hourly) and at the same temperature right up to the onset of pupariation, to determine if a hypothetical, hormonally induced, speed reduction can be observed experimentally.

Our specimens of *P. terraenovae* had an average dispersal speed of 2.36 mms^−1^ (Figure 3b), which equates to 14.16 cmmin^−1^, very much what would be expected of full‐length larvae of this species dispersing at our laboratory temperature (Charabidze et al., 2008, Figure 2). The fact that similar‐sized larvae of *C. vicina* were dispersing at the much greater rate of 4.08 mms^−1^ (=24.48 cmmin^−1^) suggests that the two species were dispersing with different strategies, the slower speed of *P. terraenovae* being consistent with the observation that larvae of *P. terraenovae* tend not to disperse far from their feeding substrate (Arnott & Turner, 2008; Byrd & Castner, 2009; Erzinçlioğlu, 1996; Mactaggart, 2018; Pohjoismäki et al., 2010).

Arthropod walking speed has been examined on the servosphere in response to odour and visual stimuli (Arnold et al., 2016), showing that changes in speed in response to changes in stimuli can be complex. For example, the average walking speed of *Rhodnius prolixus* Stål (Hemiptera: Reduviidae) increased from 30 to 45 mms^−1^ in the presence of human host metabolites, CO_2_ and NH_3_ (Otalora‐Luna et al., 2004), however the average walking speed of *Amblyomma variegatum* (Fabricius) (Ixodida: Ixodidae) decreased from 16 to 11 mms^−1^ in the presence of their pheromones (McMahon & Guerin, 2000). To our knowledge, no studies have assessed the changes in walking speed over time in the absence of external stimuli and, therefore, there are no studies with which to compare the results of our 1‐h study. The *C. vicina* larvae tested slowed down over a 1‐h period, possibly as they adapted to the new environment, but the change was mainly within the first 20 min and thereafter changes were only slight, with no pauses in activity (Figure 2). Certainly, it appears from purely visual observation of colony specimens that dispersing larvae remain active within a container until the onset of pupariation.

The negative phototaxis of calliphorid larvae is well known and it is often described as a driving force in post‐feeding dispersal, as the larvae search for a dark place prior to pupariation (Hinnemann et al., 2010), a site with reduced risk of predation or parasitisation. The results presented here show very clearly the negative phototactic behaviour of the larvae tested. The onset of negative phototactic behaviour appeared to be almost instantaneous, as all of the larval turns (approximately 90° or 180°) occurred within 5 s of the change of the light source after the start of the run. Although the behaviour was expected, it confirmed that the servosphere is a suitable apparatus on which to test the effect of external stimuli on the locomotion of apodous insects. It would be interesting to study the effect of cadaver odour stimuli on calliphorid larvae, and especially the expected change in response between feeding and post‐feeding larvae. Cadaver odours have been studied on servospheres in relation to the locomotion of newly emerged adult female carrion beetles, *Nicrophorus vespilloides* Herbst (Coleoptera: Silphidae), demonstrating higher mean walking speeds in response to odours of later decomposition stages than to early decomposition stages and controls and, also, a preference for upwind walking in the odours of the advanced decay stage (von Hoermann et al., 2013).

These results highlight the complexity of post‐feeding larval dispersal and the need for further study of this stage of blow fly development to guide forensic investigators in the collection of dispersed larvae and puparia. Post‐feeding larval dispersal is a crucial stage before pupariation and increasing our understanding of this stage of the life cycle will facilitate more accurate use of blow fly evidence in forensic investigations. Furthermore, despite the limitations of the low specimen numbers, this study has demonstrated the first successful use of a servosphere to study the crawling movement of individual fly larvae and thus opened an avenue for future experiments on these and other apodous insects, utilising this apparatus to gather both qualitative and quantitative data.

## AUTHOR CONTRIBUTIONS

**Molly Mactaggart:** Writing – original draft; investigation; conceptualization; methodology; formal analysis; writing – review and editing. **Amoret P. Whitaker:** Conceptualization; writing – review and editing. **Keith N. Wilkinson:** Writing – review and editing. **Martin J. R. Hall:** Conceptualization; writing – review and editing; methodology; formal analysis.

## CONFLICT OF INTEREST STATEMENT

The authors declare no conflicts of interest.

## Supporting information


**Video S1.** Crawling of a dry larva of *Calliphora vicina* on the apex of the servosphere. The servosphere can be seen rotating beneath the larva until the larva loses traction and falls off the ball.

**Video S2.** Crawling of a de‐ionised, water‐moistened larva of *Calliphora vicina* on the apex of the servosphere. The moist larva can be seen to retain good traction with the servosphere at all times.

## Data Availability

The data that support the findings of this study are available from the corresponding author upon reasonable request.

## References

[mve12745-bib-0001] Anderson, G.S. (2000) Minimum and maximum development rates of some forensically important Calliphoridae (Diptera). Journal of Forensic Science, 45, 824–832. 10914578

[mve12745-bib-0002] Arnold, S.E.J. , Stevenson, P.C. & Belmain, S.R. (2016) Shades of yellow: interactive effects of visual and odour cues in a pest beetle. PeerJ, 4, e2219. 27478707 10.7717/peerj.2219 PMC4950555

[mve12745-bib-0003] Arnott, S. & Turner, B. (2008) Post‐feeding larval behaviour in the blowfly, Calliphora vicina: effects on post‐mortem interval estimates. Forensic Science International, 177, 162–167. 18243615 10.1016/j.forsciint.2007.12.002

[mve12745-bib-0004] Baqué, M. , Filmann, N. , Verhoff, M.A. & Amendt, J. (2015) Establishment of developmental charts for the larvae of the blow fly *Calliphora vicina* using quantile regression. Forensic Science International, 248, 1–9. 25590766 10.1016/j.forsciint.2014.12.020

[mve12745-bib-0005] Beckstead, R.B. , Lam, G. & Thummel, C.S. (2005) The genomic response to 20‐Hydroxyecdysone at the onset of *Drosophila* metamorphosis. Genome Biology, 6, R99. 16356271 10.1186/gb-2005-6-12-r99 PMC1414087

[mve12745-bib-0006] Bell, W.J. & Kramer, E. (1980) Sex pheromone‐stimulated orientation of the American cockroach on a servosphere apparatus. Journal of Chemical Ecology, 6, 287–295.

[mve12745-bib-0007] Benecke, M. (2005) Arthropods and corpses. Forensic Pathology Reviews, 2, 207–240.

[mve12745-bib-0008] Bugelli, V. , Campobasso, C.P. , Verhoff, M.A. & Amendt, J. (2017) Effects of different storage and measuring methods on larval length values for the blow flies (Diptera: Calliphoridae) *Lucilia sericata* and *Calliphora vicina* . Science and Justice, 57, 159–164. 28454623 10.1016/j.scijus.2016.10.008

[mve12745-bib-0009] Byrd, J. & Castner, J. (2009) Forensic entomology: the utility of arthropods in legal investigations, 2nd edition. Boca Raton, FL: CRC Press.

[mve12745-bib-0010] Charabidze, D. , Bourel, B. , Leblanc, H. , Hedouin, V. & Gosset, D. (2008) Effect of body length and temperature on the crawling speed of *Protophormia terraenovae* larvae (Robineau‐Desvoidy) (Diptera Calliphoridae). Journal of Insect Physiology, 54, 529–533. 18222465 10.1016/j.jinsphys.2007.11.010

[mve12745-bib-0043] Donovan, S.E. , Hall, M.J.R, Turner, B.D. & Moncrieff, C.B. (2006) Larval growth rates of the blow fly, Calliphora vicina, over a range of temperatures. Medical and Veterinary Entomology, 20, 106–114. 16608495 10.1111/j.1365-2915.2006.00600.x

[mve12745-bib-0011] Erzinçlioğlu, Z. (1996) Blowflies (Naturalists' handbook 23), 1st edition. Slough: Richmond Publishing Co Ltd.

[mve12745-bib-0012] Gomes, L. , Godoy, W.A.C. & von Zuben, C.J. (2006) A review of postfeeding dispersal in blowflies: implications for forensic entomology. Naturwissenschaften, 93, 207–215. 16538375 10.1007/s00114-006-0082-5

[mve12745-bib-0014] Gomes, L. , Sanches, M.R. & von Zuban, C.J. (2005) Dispersal and burial behaviour in larvae of *Chrysomya megacephala* and *Chrysomya albiceps* (Diptera, Calliphoridae). Journal of Insect Behaviour, 18, 281–292.

[mve12745-bib-0015] Gomes, L. , Sanches, M.R. & Von Zuben, C.J. (2007) Behaviour of the combined radial post‐feeding larval dispersal of the blowflies *Chrysomya megacephala* and *Chrysomya albiceps* (Diptera: Calliphoridae) and implications for forensic entomology. Brazilian Archives of Biology and Technology, 50, 279–288.

[mve12745-bib-0016] Grassberger, M. & Reiter, C. (2002) Effect of temperature on development of the forensically important holarctic blow fly *Protophormia terraenovae* (Robineau‐Desvoidy) (Diptera: Calliphoridae). Forensic Science International, 128, 177–182. 12175962 10.1016/s0379-0738(02)00199-8

[mve12745-bib-0017] Hinnemann, A. , Niederegger, S. , Hanslik, U. , Heinzel, H.G. & Spieß, R. (2010) See the light: electrophysiological characterization of the Bolwig organ's light response of *Calliphora vicina* 3rd instar larvae. Journal of Insect Physiology, 56, 1651–1658. 20603127 10.1016/j.jinsphys.2010.06.010

[mve12745-bib-0018] Hodecek, J. , Fumagalli, L. & Jakubec, P. (2024) All insects matter: a review of 160 entomology cases from 1993 to 2007 in Switzerland—part 1 (Diptera). Journal of Medical Entomology, 61, 400–409. 38157316 10.1093/jme/tjad164 PMC10936168

[mve12745-bib-0019] Kočárek, P. (2001) Diurnal patterns of postfeeding dispersal in carrion blowflies (Diptera: Calliphoridae). European Journal of Entomology, 98, 117–119.

[mve12745-bib-0020] Kramer, E. (1976) The orientation of walking honeybees in odour fields with small concentration gradients. Physiological Entomology, 1, 27–37.

[mve12745-bib-0021] Lewis, A. & Benbow, M. (2011) When entomological evidence crawls away: *Phormia regina* en masse larval dispersal. Journal of Medical Entomology, 48, 1112–1119. 22238869 10.1603/me11093

[mve12745-bib-0022] Mactaggart, M.M. (2018) The post‐feeding larval dispersal of forensically important UK blow flies. PhD thesis, University of Winchester, UK, 201 pp.

[mve12745-bib-0023] McMahon, C. & Guerin, P. (2000) Responses of the tropical bont tick, *Amblyomma variegatum* (Fabricius), to its aggregation‐attachment pheromone presented in an air stream on a servosphere. Journal of Comparative Physiology, 186, 95–103. 10659046 10.1007/s003599900064

[mve12745-bib-0025] Nagaya, N. , Mizumoto, N. , Abe, M. , Dobata, S. , Sato, R. & Fukisawa, R. (2017) Anomalous diffusion on the servosphere: a potential tool for detecting inherent organismal movement patterns. PLoS One, 12, e0177480. 28570562 10.1371/journal.pone.0177480 PMC5453419

[mve12745-bib-0027] Otalora‐Luna, F. , Perret, J.‐L. & Guerin, P. (2004) Appetence behaviours of the Triatomine bug *Rhodinius prolixus* on a servosphere in response to the host metabolites carbon dioxide and ammonia. Journal of Comparative Physiology A: Neuroethology, Sensory, Neural, and Behavioral Physiology, 190, 847–854. 15503053 10.1007/s00359-004-0540-5

[mve12745-bib-0026] Otálora‐Luna, F. & Dickens, J. (2011) Spectral preference and temporal modulation of photic orientation by Colorado potato beetle on a servosphere. Entomologia Experimentalis et Applicata, 138, 93–103.

[mve12745-bib-0028] Pohjoismäki, J.L.O. , Karhunen, P.J. , Goebeler, S. , Saukko, P. & Sääksjärvi, I.E. (2010) Indoors forensic entomology: colonization of human remains in closed environments by specific species of sarcosaprophagous flies. Forensic Science International, 199, 38–42. 20304573 10.1016/j.forsciint.2010.02.033

[mve12745-bib-0029] Rickli, M. , Guerin, P. & Diehl, P. (1992) Palmitic acid released from honeybee worker larvae attracts the parasitic mite *Varroa jacobsoni* on a servosphere. Naturwissenschaften, 79, 320–322.

[mve12745-bib-0031] Robinson, L.A. , Bryson, D. , Bulling, M.T. , Sparks, N. & Wellard, K.S. (2018) Post‐feeding activity of *Lucilia sericata* (Diptera: Calliphoridae) on common domestic indoor surfaces and its effect on development. Forensic Science International, 286, 177–184. 29579718 10.1016/j.forsciint.2018.03.010

[mve12745-bib-0032] Sakuma, M. (2002) Virtual reality experiments on a digital servosphere: guiding male silkworm moths to a virtual odour source. Computers and Electronics in Agriculture, 35, 243–254.

[mve12745-bib-0033] Salanitro, L.B. , Massaccesi, A.C. , Urbisaglia, S. , Pería, M.E. , Centeno, N.D. & Chirino, M.G. (2022) *Calliphora vicina* (Diptera: Calliphoridae): growth rates, body length differences, and implications for the minimum post‐mortem interval estimation. Revista de la Sociedad Entomológica Argentina, 81, 39–48.

[mve12745-bib-0035] Taneja, J. & Guerin, P. (1995) Oriented responses of the Triatomine bugs *Rhodnius prolixus* and *Triatoma infestans* to vertebrate odours on a servosphere. Journal of Comparative Physiology A: Neuroethology, Sensory, Neural, and Behavioral Physiology, 176, 455–464.

[mve12745-bib-0036] Thummel, C. (1996) Flies on steroids—*Drosophila* metamorphosis and the mechanisms of steroid hormone action. Trends in Genetics, 12, 306–310. 8783940 10.1016/0168-9525(96)10032-9

[mve12745-bib-0037] Turpin, C. , Kyle, C. & Beresford, D.V. (2014) Postfeeding larval dispersal of late season blow flies (Calliphoridae) in southern Ontario, Canada. Journal of Forensic Sciences, 59, 1295–1302. 24602116 10.1111/1556-4029.12469

[mve12745-bib-0038] Von Hoermann, C. , Steiger, S. , Müller, J.K. & Ayasse, M. (2013) Too fresh is unattractive! The attraction of newly emerged *Nircrophorus vespilloides* females to odour bouquets of large cadavers at various stages of decomposition. PLoS One, 8, e58524. 23516497 10.1371/journal.pone.0058524 PMC3596307

[mve12745-bib-0039] Ward, R.E. , Reid, P. , Bashirullah, A. , D'Avino, P.P. & Thummel, C.S. (2003) GFP in living animals reveals dynamic developmental responses to ecdysone during *Drosophila* metamorphosis. Developmental Biology, 256, 389–402. 12679111 10.1016/s0012-1606(02)00100-8

[mve12745-bib-0040] Ware, C. , Lowe, M. , Sivell, D. , Baker, A. , Bantock, T. , Barclay, M. et al. (2016) Further developments of the flora and fauna of the Wildlife Garden at the Natural History Museum, London: twenty years of species recording. The London Naturalist, 95, 45–159.

[mve12745-bib-0041] Yasushi, I. , Ogawa, H. , Shidara, H. , Sakura, M. , Sato, T. , Hojo, M. et al. (2019) Markerless visual servo control of a servosphere for behavior observation of a variety of wandering animals. Advanced Robotics, 33, 183–194.

[mve12745-bib-0042] Zdarek, J. , Rohlf, R. , Blechl, J. & Fraenkel, G. (1981) A hormone effecting immobilization in pupariating fly larvae. Journal of Experimental Biology, 93, 51–63.

